# Transposition of Great Arteries: New Insights into the Pathogenesis

**DOI:** 10.3389/fped.2013.00011

**Published:** 2013-06-06

**Authors:** Marta Unolt, Carolina Putotto, Lucia M. Silvestri, Dario Marino, Alessia Scarabotti, Valerio Massaccesi, Angela Caiaro, Paolo Versacci, Bruno Marino

**Affiliations:** ^1^Department of Pediatrics, “Sapienza” University of Rome, Rome, Italy; ^2^Eleonora Lorillard Spencer Cenci Foundation, Rome, Italy

**Keywords:** transposition of great arteries, heterotaxy, genetics of congenital heart diseases, embryology of congenital heart diseases, experimental animal models

## Abstract

Transposition of great arteries (TGA) is one of the most common and severe congenital heart diseases (CHD). It is also one of the most mysterious CHD because it has no precedent in phylogenetic and ontogenetic development, it does not represent an alternative physiological model of blood circulation and its etiology and morphogenesis are still largely unknown. However, recent epidemiologic, experimental, and genetic data suggest new insights into the pathogenesis. TGA is very rarely associated with the most frequent genetic syndromes, such as Turner, Noonan, Williams or Marfan syndromes, and in Down syndrome, it is virtually absent. The only genetic syndrome with a strong relation with TGA is Heterotaxy. In lateralization defects TGA is frequently associated with asplenia syndrome. Moreover, TGA is rather frequent in cases of isolated dextrocardia with situs solitus, showing link with defect of visceral situs. Nowadays, the most reliable method to induce TGA consists in treating pregnant mice with retinoic acid or with retinoic acid inhibitors. Following such treatment not only cases of TGA with d-ventricular loop have been registered, but also some cases of congenitally corrected transposition of great arteries (CCTGA). In another experiment, the embryos of mice treated with retinoic acid in day 6.5 presented Heterotaxy, suggesting a relationship among these morphologically different CHD. In humans, some families, beside TGA cases, present first-degree relatives with CCTGA. This data suggest that monogenic inheritance with a variable phenotypic expression could explain the familial aggregation of TGA and CCTGA. In some of these families we previously found multiple mutations in laterality genes including Nodal and ZIC3, confirming a pathogenetic relation between TGA and Heterotaxy. These overall data suggest to include TGA in the pathogenetic group of laterality defects instead of conotruncal abnormalities due to ectomesenchymal tissue migration.

## Introduction

Transposition of the great arteries (TGA) is one of the most common and severe, but also one of the most mysterious, congenital heart diseases (CHD).

With a prevalence of 3,54/10,000 live births in Europe, it is the fourth most common type of major cardiac defect (1), representing 5% of all CHD and 34% of conotruncal defects with situs solitus (2).

It is a severe CHD: indeed, if not treated, it is the leading cause of cardiac death in neonates and infants (3).

Last but not least, it is still a rather mysterious CHD: in phylogenetic and ontogenetic development it has no precedent (4); it does not represent an alternative physiological model of blood circulation (5); its etiology and its morphogenesis are still largely unknown (2).

Over the last years, great improvements have been made in diagnosis, as well as in medical and surgical treatment of this CHD (5–10). As a consequence, nowadays, the overall survival of these patients is significantly better (11). In this review we report on the recent genetic and embryological researches on this fascinating CHD.

## Pathogenetic Classification

In 1986 Clark (12) introduced a pathogenetic classification of CHDs, that has been commonly accepted (13). This classification consists of six causative mechanism of CHDs: (i) ectomesenchymal tissue migration abnormalities; (ii) intracardiac blood flow defects; (iii) cell death abnormalities; (iv) extra cellular matrix abnormalities; (v) abnormal targeted growth; (vi) anomalies of visceroatrial situs and ventricular looping. TGA, classically considered as a conotruncal defect, according to Clark’s classification was considered an anomaly of ectomesenchymal tissue migration.

On the other hand, in the Baltimore–Washington Infant Study, a fundamental epidemiologic investigation of CHD, Ferencz et al. showed that extracardiac anomalies had different prevalence in TGA (10%, mostly kidney and cerebral anomalies) in comparison with other conotruncal defects (35%), such as tetralogy of Fallot, truncus arteriosus, and interrupted aortic arch, which are frequently associated with DiGeorge syndrome and del22q11 (2, 14). Moreover, in this study TGA resulted more common in males than females. So the Authors suggested to consider TGA as a CHD etiologically different than others conotruncal defect (2).

## Embryologic Theories

There are two main theories which try to explain the embryological mechanisms of TGA.

One theory, formulated by Goor and Edwards (15, 16), suggests that TGA is caused by the lack of the normal, clockwise (when the heart is viewed from above), rotation of the aorta toward the left ventricle. This defect of infundibular rotation is supposed to be caused by an abnormal resorption or underdevelopment of the subpulmonary conus with an abnormal persistence of the subaortic conus. Therefore, according to this theory, TGA is an extreme case in the spectrum of “dextroposition of the aorta” that goes from various forms of double outlet right ventricle, through tetralogy of Fallot, up to malalignment type of ventricular septal defects (17).

The second theory, proposed by de la Cruz (18, 19), focuses on abnormal spiraling of the aorto-pulmonary septum. She suggested that in the embryogenesis, either normal or pathological, there is no rotation at the infundibular level. TGA is due, instead, to a linear rather than spiral development of aorto-pulmonary septum, that puts the forth aortic arch (the future aorta) in contact with the anterior conus, situated on the right ventricle.

There are arguments both in favor and against each of these theories.

The “infundibular theory” seems to explain better cases of TGA with ventricular septal defect and with a certain degree of pulmonary overriding, which are morphologically similar to the double outlet right ventricle. It is less helpful, though, in explaining cases with intact ventricular septum.

On the other hand, the “extracardiac theory” does not account for the great variability of infundibular morphology in this cardiac defect (20, 21).

**Figure 1:**
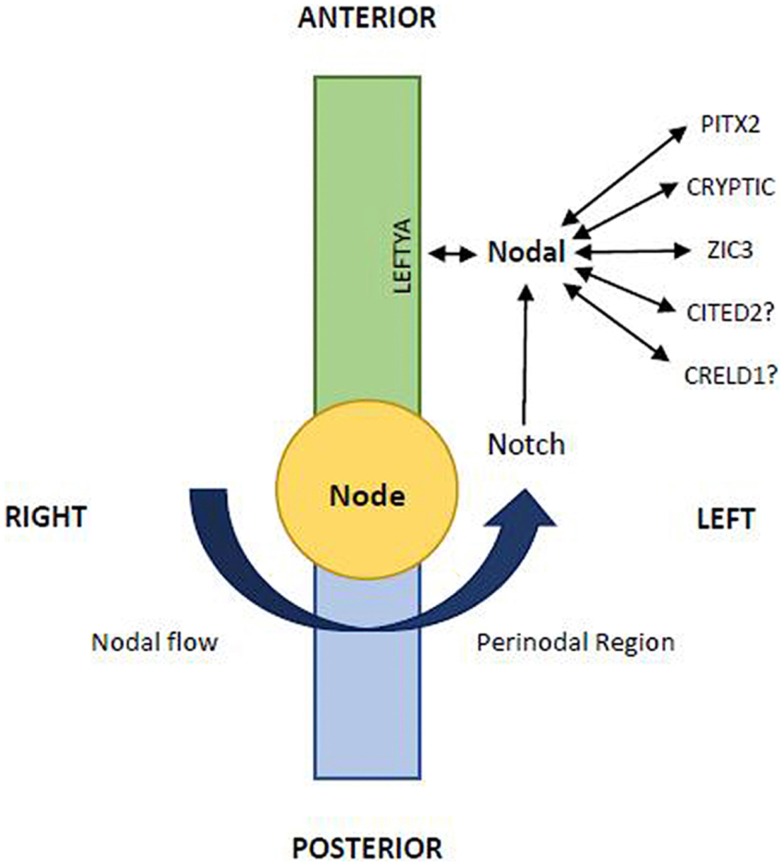
**The L–R pattering is caused at the node by an early breaking of bilateral symmetry**. The nodal gene is essential in this function and the midline acts as a physical and molecular barrier to determine correct side-specific gene expression. The leftward Nodal flow (arrow) transport to the left wall of the node the nodal vesicular parcels. At this level Nodal interplays with other signalings including Notch, LeftyA, Cryptic, Pitx2, etc. (Modified from Zhu et al 2006; 14:14–25)

However, a recent study showed that a spiraling migration (clockwise when viewed from above) of cells from the right and left secondary heart field is necessary for the elongation and a proper alignment of the pulmonary outflow tract, so that it may acquire its right-handed spiral pattern (22, 23). In 2006, Bajolle et al. (24) demonstrated the occurrence in Pitx2 mutant embryos of conotruncal defects with rotational anomalies, including TGA (Figure 1), which confirms the importance of the spiral movement of outflow tract (18, 19).

## Genetic Syndromes and Extracardiac Malformations

Transposition of great arteries is very rarely associated with the most frequent genetic syndromes, such as Turner, Noonan, Williams or Marfan syndromes (Table 1), and in Down syndrome, it is virtually absent (25). It is interesting to note that TGA might be sporadically associated with trisomy 8 and 18, with VACTERL and CHARGE syndromes (2, 14), as well as with tuberous sclerosis (26), deletion of the long arm of chromosome 11 (27) and of the short arm of chromosome 18 (28) (Table 1). Moreover, in our patients with TGA, we have observed also isolated cases of anomalies of chromosome 3, 15, X (unpublished data) (Table 1).

**Table 1:** **Genetic and non-genetic causes of TGA**.

Genetic	Syndromic	Heterotaxy (right isomerism) (35)
		Trisomy 8 (2, 14)
		Trisomy 18 (2, 14)
		VACTERL (2, 14)
		CHARGE (2, 14)
		Tuberous sclerosis (26)
		Deletion 11q (27)
		Deletion 18p (28)
		Anomalies chromosome 3, 15, X (unpublished data)
		DiGeorge/deletion 22q11 (14, 29–33)
		Turner syndrome (25)
		Noonan syndrome (25)
		Williams syndrome (25)
		Marfan syndrome (25)
	Non-syndromic	ZIC3 (41–43, 45)
		Nodal (44, 47)
		CFC1 (45, 46)
		Smad2 (44)

Teratogens		Maternal diabetes (56, 57)
		Maternal infections (14)
		Ionizing radiations (14)
		Pesticides (52)
		Ibuprofen (14)
		Antiepileptic drugs (53)
		Hormonal drugs (54)
		Other drugs (55)
		*In vitro* fertilization (58)

Furthermore, extracardiac anomalies are extremely rare in TGA patients, and include mostly kidney diseases and cerebral abnormalities (2).

The association of TGA with DiGeorge/Velocardiofacial Syndrome and with del22q11 is a topic that deserves wider discussion. Patient with DiGeorge syndrome may present TGA (14, 29), as well as patient with del22q11 (30–33) (Table 1). Animal experiments have demonstrated that the ablation of neural crest in chick embryos may results in TGA (34). Nevertheless TGA cannot be considered a characteristic cardiac defect of del22q11 syndrome, unlike such defects as tetralogy of Fallot, truncus arteriosus, and interrupted aortic arch type B (33). Instead only 1% of patient with TGA have del22q11. Thus it is possible to hypothesize a pathogenetic relationship between TGA and del22 but their association should be considered rare and sporadic (30–33).

The only genetic syndrome with a strong relation with TGA is the Heterotaxy (Table 1). First of all, TGA is rather frequent in cases of isolated dextrocardia with situs solitus, showing link with defect of visceral situs (35). Moreover, in lateralization defects (Heterotaxy or isomerisms) TGA is frequently associated with complete atrioventricular canal (CAVC), mostly in asplenia syndrome (right isomerism). TGA associated with CAVC has been reported in almost 100% of cases of asplenia syndrome (35, 36), with d-ventricular loop {A,D,D}as well as with l-ventricular loop {A,L,L}. On the other hand, it is interesting to note that TGA is significantly rarer in polysplenia syndrome (left isomerism): in these cases great arteries are usually normally related {A,D,S}or “inversely” normally related {A,L,I}(35, 37). This different prevalence has statistical significance (35–37), and has to be given a pathogenetic explanation.

Also in experimental animal models of Heterotaxy syndrome, TGA has been frequently reported, both in d-ventricular loop and in l-ventricular loop (38–40). It is worth noting that in some large families with recurrence of Heterotaxy and ZIC3 gene mutation (41–43) (Table 1), besides cases with situs inversus, with polysplenia or asplenia, there are cases of congenitally corrected transposition of great arteries (CCTGA) with situs solitus {S,L,L}. Therefore, we can suppose, therefore, that the same genetic mechanism could produce variable phenotypes in these families, including not only different kinds of Heterotaxy (asplenia or polysplenia), but also situs solitus CCTGA. Moreover, in mice knockout of Smad2 and Nodal gene (genes involved in the process of laterality determination) leads to TGA associated with right pulmonary isomerism of the lung, in more than 50% of cases (44) (Table 1). Finally, in some patients with isolated or syndromic TGA have been observed mutations of ZIC3 (45), CFC1 (45, 46), and Nodal (47) (Table 1). All these are “laterality genes” associated with Heterotaxy (Figure 1).

A possible relationship between TGA and anomalies of ventricular looping has been already speculated in times past (1, 48–50), but scientific literature has not supported this hypothesis (51). Nowadays, the recent evidences suggest that TGA is most closely associated with the Heterotaxy syndrome.

## Teratogens and Experimental Animal Models

An important issue, in the etiological and epidemiological studies of the TGA, is the occurrence of this CHD due to intake of teratogens, especially pesticides, by mothers (52) (Table 1). Cases of TGA associated with intake of antiepileptic (53), hormonal (54), and other drugs (55) are rarely reported, while the prevalence of TGA is higher in infants of diabetic mothers (56, 57) and as result of maternal infection (such as flu), intake of ibuprofen or ionizing radiation (14), as well as in cases of *in vitro* fertilization (58) (Table 1). A reduced occurrence of CHD, including TGA, has been reported as result of periconceptional intake of folic acid, which may be considered a protective factor against congenital malformations (59).

To induce TGA with teratogens in experimental animal models is quite difficult. Initially radiations and trypan blue were used, but now the most reliable method consist in treating pregnant mice with retinoic acid (1, 38). It is interesting to note that following such treatment not only cases of TGA with d-ventricular loop have been registered, but also some cases of TGA with l-ventricular loop (CCTGA). In another experiment, the embryos of mice treated with retinoic acid in days 6.5 presented Heterotaxy. We could explain the differences in the cardiac phenotype thus obtained with a different timing of teratogen treatment (1, 38). This notwithstanding, the pathogenetic mechanism seems to be the same, suggesting that there is a relationship among these morphologically different cardiac defects.

We recently obtained TGA by administration of a retinoic acid competitive antagonist in pregnant mice (60), showing that critical levels of retinoic acid must be present for normal heart and conotruncal development. These teratogenic effects may be consistently reduced by folic acid and methionine supplementation (61). Based on these data and following the results of further experiments, we suggested that the teratogenic development of TGA was due to Hif1α down-regulation in response to blocking retinoic acid (62). Hif1α has an essential role in cardiac embryology and one of his downstream target Cited2 is involved in left/right determination (Figure 1). Overall these results confirm a pathogenetic links between TGA and lateralization defects with Heterotaxy.

## Familial Recurrence

Usually TGA is considered to have a low risk of familial recurrence. The English multicentric study by Burn et al. reported no familial cases of TGA (63). Our experience on this topic is rather different (64–66): in a multicentric Italian study the recurrence rate in siblings of patients with TGA were calculated at 1.7% (66). It is interesting to underline that in some families, beside TGA {S,D,D}cases, there were first-degree relatives (siblings or parents) with CCTGA {S,L,L}. This data suggest that monogenic inheritance (autosomal dominant or recessive) with a variable phenotypic expression could explain the familial aggregation of TGA and CCTGA. In some of these families we found multiple mutations in laterality genes including Nodal and ZIC3 (Figure 1), confirming a pathogenetic relation between TGA and Heterotaxy (67).

## Ventricular Looping and Spirality, Heterotaxy, and Transposition

Ventricular looping is genetically determined in all vertebrates including humans, and it represents the first morphological sign of L–R asymmetry in embryonic development (68).

In normal embryogenesis the rightward looping of the heart causes d-ventricular loop, continues with a looping movement that brings the right ventricle anteriorly, and finally involves the infundibula and the great arteries in a “rightward spiralization.” As a result of this movement the ventricles, infundibula, and great arteries fall into their regular position. The “rightward spiralization” of the heart represents a pattern corresponding also with the normal rightward rotation of the bowel (69) and the normal cerebral asymmetry (70, 71).

**Figure 2:**
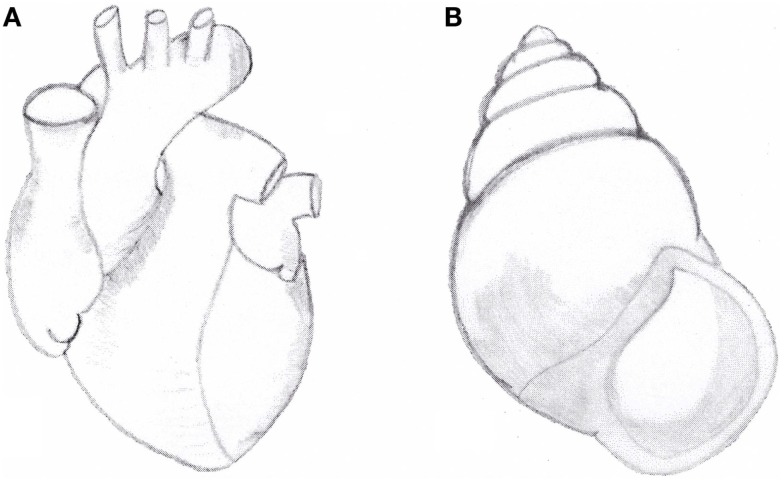
**(A)** Anatomical aspect of normal heart with situs solitus. Note the right-handed spiral pattern of outflow tract and great arteries. **(B)** Right-handed shell of the snail *Amphidromus perversus rufocinctus*.

It is remarkable that the same rightward spiralization is prevalent, though not exclusive, in the development of other organisms such as shells (72) (Figure 2), some bacteria including *Bacillus subtilis* (73) and some climber plant including *Convolvulus arvensis* (74).

We have previously suggested that the normal (right-handed) spiral pattern of the great arteries and the prevailing right-handed spiral pattern of snail shells show some phenotypic similarities (35, 75–77) (Figure 2).

Our morphological observations of cardiac defects in laterality disorders, including TGA, showed that in persons with situs inversus, the normal (right-handed) spiral pattern of the great arteries is inverted, showing a left-handed spiral pattern similar to a minority of shells. On the other hand, in subjects with TGA with or without asplenia/right isomerism any spiral pattern of the great arteries is lost: the two great arteries run parallel to each other, without any sign of spiralization (35, 75, 76). Therefore, we hypothesized that these normal and abnormal anatomical aspects, comparable in humans and in shells, could share a common genetic mechanism (35, 75, 77).

Recent articles confirmed our suggestion showing the role of Nodal signaling in left-right asymmetry in snails: embryos of dextral (right-handed) species *Lottia gigantea* express Nodal gene on their right side, while embryos of sinistral (left-handed) species *Biomphalaria glabrata* express Nodal on their left side (78, 79). As in vertebrates the heart designates the situs, in snails the pattern of chirality of the shell (right-handed vs. left-handed) is a sign of situs and of internal organ arrangement (80–82). Moreover, the recent study of Grande and Patel showed that pharmacologic inhibition of the Nodal pathway produces loss of shell chirality, which results in a straight non-spiralized shell (79). Interestingly, it recalls the straight non-spiral phenotype of the great arteries in human TGA in some cases, associated of mutation of the same Nodal gene (67, 80–82). We can conclude that Nodal gene (Figure 1), strongly conserved by phylogenetic mechanisms, is a gene of development involved in the morphogenetic mechanism of normal and abnormal spiralization of great arteries of vertebrates and of normal and abnormal spiralization of the snail shells (78–82).

Moreover, what still needs to be elucidated is the possible relationship between the spiralization of the cardiac outflow tract and of great arteries and the hypothesis of spiralized pattern of myocardial band (77, 83–85), the chirally asymmetric paths of intracardiac flow (86, 87), and the spiral pattern detected at cellular and molecular level (83, 87).

## Conclusion

In 1998, Brett Casey, one of the pioneers in the field of genetics of Heterotaxy, asked “Are some complex, isolated heart malformations actually unrecognized manifestations of aberrant left–right asymmetry development? (43).” Nowadays, the overall epidemiological, experimental, and genetic data suggest that TGA, even in situs solitus, can be expression of laterality defects, as it has already been shown for some forms of CAVC (88–91).

Even though the detailed pathogenesis of TGA remains rather mysterious, maybe there are some gleams of light in relation of normal or abnormal spiralization and lateralization mechanisms.

## Conflict of Interest Statement

The authors declare that the research was conducted in the absence of any commercial or financial relationships that could be construed as a potential conflict of interest.
